# Supplementary material for: Dysregulation of Macropinocytosis Processes in Glioblastomas May Be Exploited to Increase Intracellular Anti-Cancer Drug Levels: The Example of Temozolomide
Source: Cancers (Basel). 2019 Mar 22;11(3):411. doi: 10.3390/cancers11030411 (PMC6468498; doi:10.3390/cancers11030411)
Supplement: Supplementary file 1 [file cancers-11-00411-s001.pdf]

cancers

Supplementary Materials: Dysregulation of Macropinocytosis Processes in Glioblastomas May Be Exploited to Increase Intracellular Anti-Cancer Drug Levels: The Example of Temozolomide

Margaux Colin, Cédric Delporte, Rekin's Janky, Anne-Sophie Lechon, Gwendoline Renard, Pierre Van Antwerpen, William A. Maltese and Véronique Mathieu

Table S1. Statistical comparison of mRNA expression data of macropinocytosis gene probes in the GSE53733 dataset.

Gene	Name	Number of Probe	Kruskall-Wallis	2 Tailed
EGFR	Epidermal growth factor receptor	8	NS/NS/NS/NS/NS/*/NS/NS	0/0/0/0/0/1/0/0
PDGFRA	Platelet derived growth factor receptor alpha	3	NS/*/NS	0/1/0
PDGFRB	Platelet derived growth factor receptor beta	1	NS	0
H-RAS	H-Ras proto-oncogene, GTPase	1	NS	0
K-RAS	K-RAS proto-oncogene, GTPase	5	NS/NS/NS/NS/NS	0/0/0/0/0
CDC42	Cell division cycle 42	5	NS/NS/NS/NS/NS	0/0/0/0/0
RAC1	Ras-related C3 botulinum toxin substrate 1 (rho family, small GTP binding protein Rac1)	4	NS/NS/NS/NS	0/0/0/0
GIT1	GIT ArfGAP 1	1	NS	0
ARF1	ADP ribosylation factor 1	4	NS/NS/NS/NS	0/0/0/0
ARF6	ADP ribosylation factor 6	3	NS/NS/NS	0/0/0
PAK1	p21 (RAC1) activated kinase 1	4	NS/NS/NS/NS	0/0/0/0
WASF1	WAS protein family member 1	1	NS	0
CYFIP1	Cytoplasmic FMR1 interacting protein 1	1	**	1
NCKAP1	NCK associated protein 1	2	NS/NS	0/0
ABI1	Abl-interactor 1	2	NS/*	0/1
BRK1	BRICK1, SCAR/WAVE actin nucleating complex subunit	2	NS/NS	0/0
ARPC1A	Actin related protein 2/3 complex subunit 1A	1	NS	0
ARPC1B	Actin related protein 2/3 complex subunit 1B	1	NS	0
ARPC2	Actin related protein 2/3 complex subunit 2	3	NS/NS/NS	0/0/0
ARPC3	Actin related protein 2/3 complex subunit 3	1	NS	0

ARPC4	Actin related protein 2/3 complex subunit 4	2	NS/NS	0/0
ARPC5	Actin related protein 2/3 complex subunit 5	4	NS/*/NS/NS	0/1/0/0
SWAP70	SWAP switching B-cell complex 70kDa subunit	3	**/**/*	1/1/1
RAB34	RAB34, member RAS oncogene family	2	NS/NS	0/0
CTBP1	C-terminal binding protein 1	3	NS/NS/NS	0/0/0
PLD1	Phospholipase D1	6	NS/NS/NS/NS/NS/NS	0/0/0/0/0/0
RAB5A	RAB5A, member RAS oncogene family	3	NS/NS/NS	0/0/0
RAB5B	RAB5B, member RAS oncogene family	1	NS	0
RAB5C	RAB5C, member RAS oncogene family	2	NS/NS	0/0
RAB20	RAB20, member RAS oncogene family	1	NS	0
RAB21	RAB21, member RAS oncogene family	4	NS/NS/NS/NS	0/0/0/0
RAB7A	RAB7A, member RAS oncogene family	6	NS/NS/NS/NS/NS/NS	0/0/0/0/0/0
RAB7B	RAB7B, member RAS oncogene family	2	NS/NS	0/0
LAMP1	Lysosomal associated membrane protein 1	3	NS/NS/NS	0/0/0
SNX1	Sorting nexin 1	3	NS/NS/NS	0/0/0
SNX5	Sorting nexin 5	5	NS/NS/NS/NS/NS	0/0/0/0/0
RAB11A	RAB11A, member RAS oncogene family	3	NS/NS/NS	0/0/0
RAB11B	RAB11B, member RAS oncogene family	2	NS/NS	0/0

This dataset regroups 70 samples of GBM patients with different overall survivals (16 samples of short survival < 12 months; 23 samples with long survival > 36 months and 31 samples of intermediate survival). Kruskall-Wallis results are provided per probe (probe 1/ probe 2/…). NS: $p > 0.05$; *: $p < 0.05$; **: $p < 0.01$; ***: $p < 0.001$. The number of 2-tailed significant results per probe is then provided in a similar way: e.g., 0/1/0 means for probe 1 that no 2-tailed comparison reached significant threshold ($p < 0.05$), for probe 2, one 2-tailed comparison turned out significant, and again no significance was found between groups for probe 3. Note that the maximum comparison number is 3.

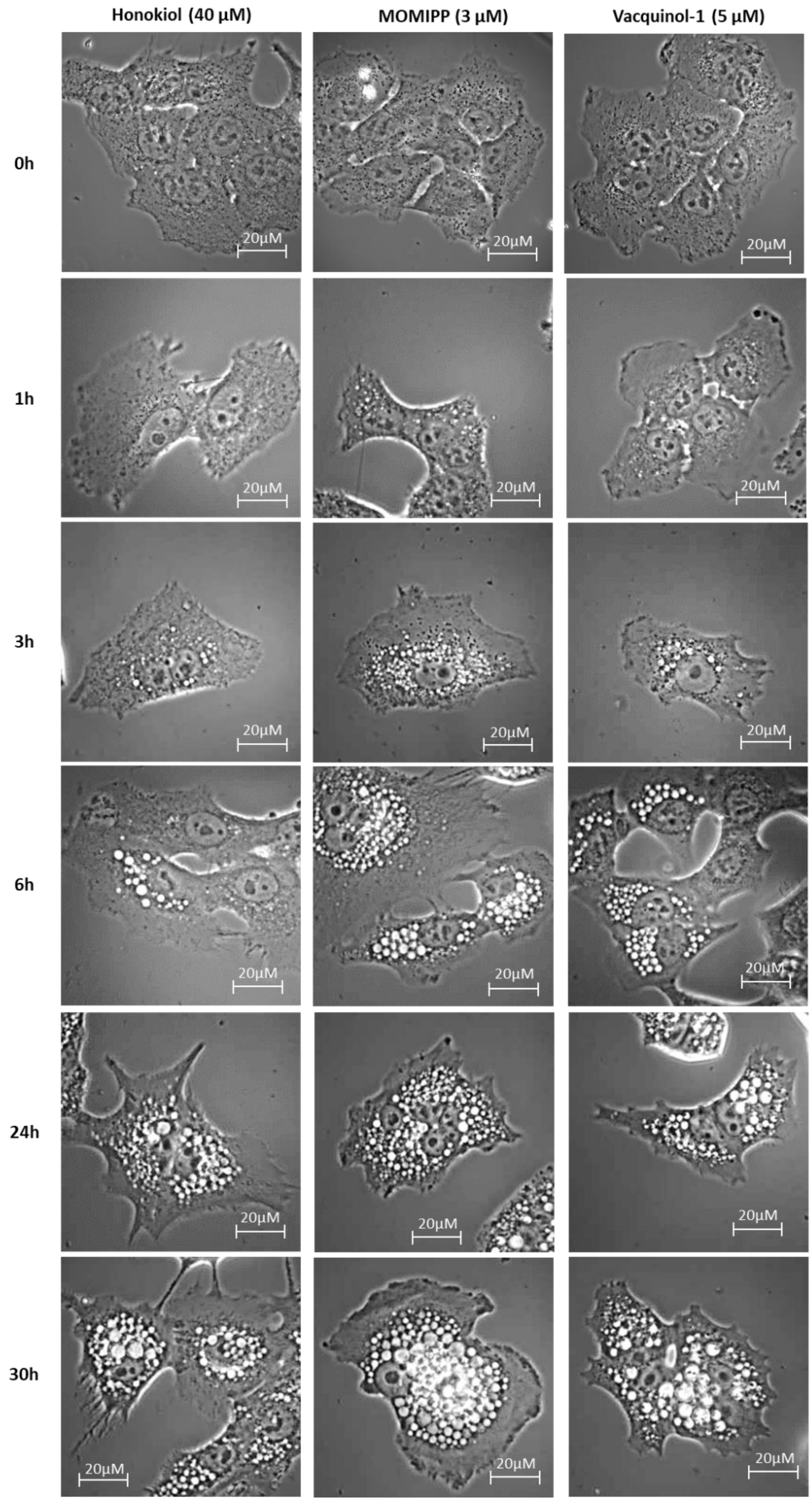

Figure S1. Morphological illustration of the vacuoles formed after treatment of Hs683 cell line with honokiol (40 μM), MOMIPP (3 μM) or vacquinol-1 (5 μM) over time. Vacuoles appear after 6 h of treatment with honokiol, 3 h with vacquinol-1 and only 1 h of treatment with MOMIPP. The experiment has been conducted two times in triplicates.

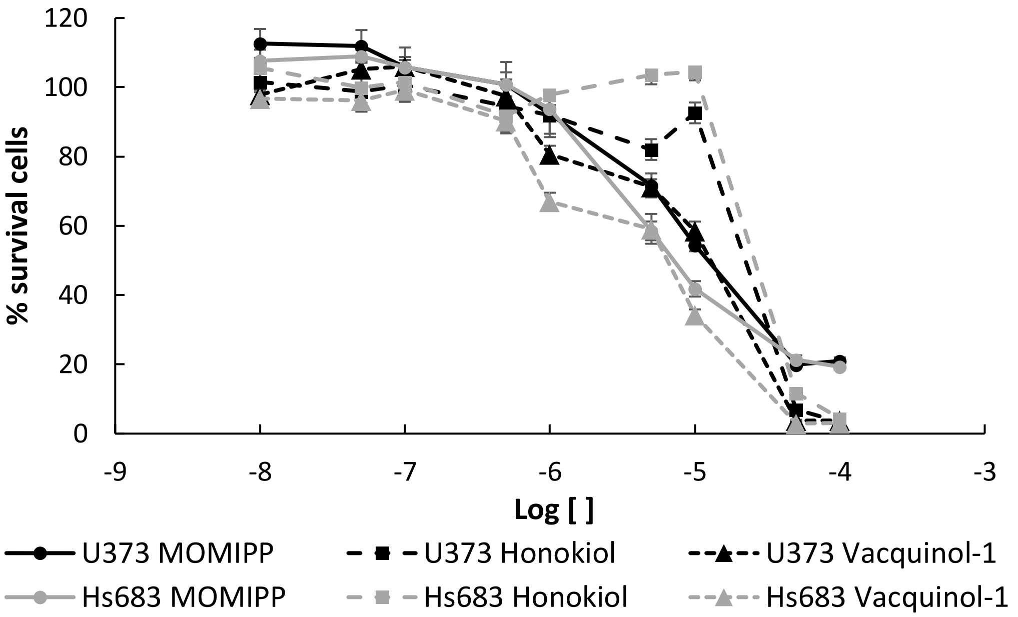

Figure S2. MTT curves of the three products used in this study. Data are expressed as mean ± SEM of two experiments made in six replicates.

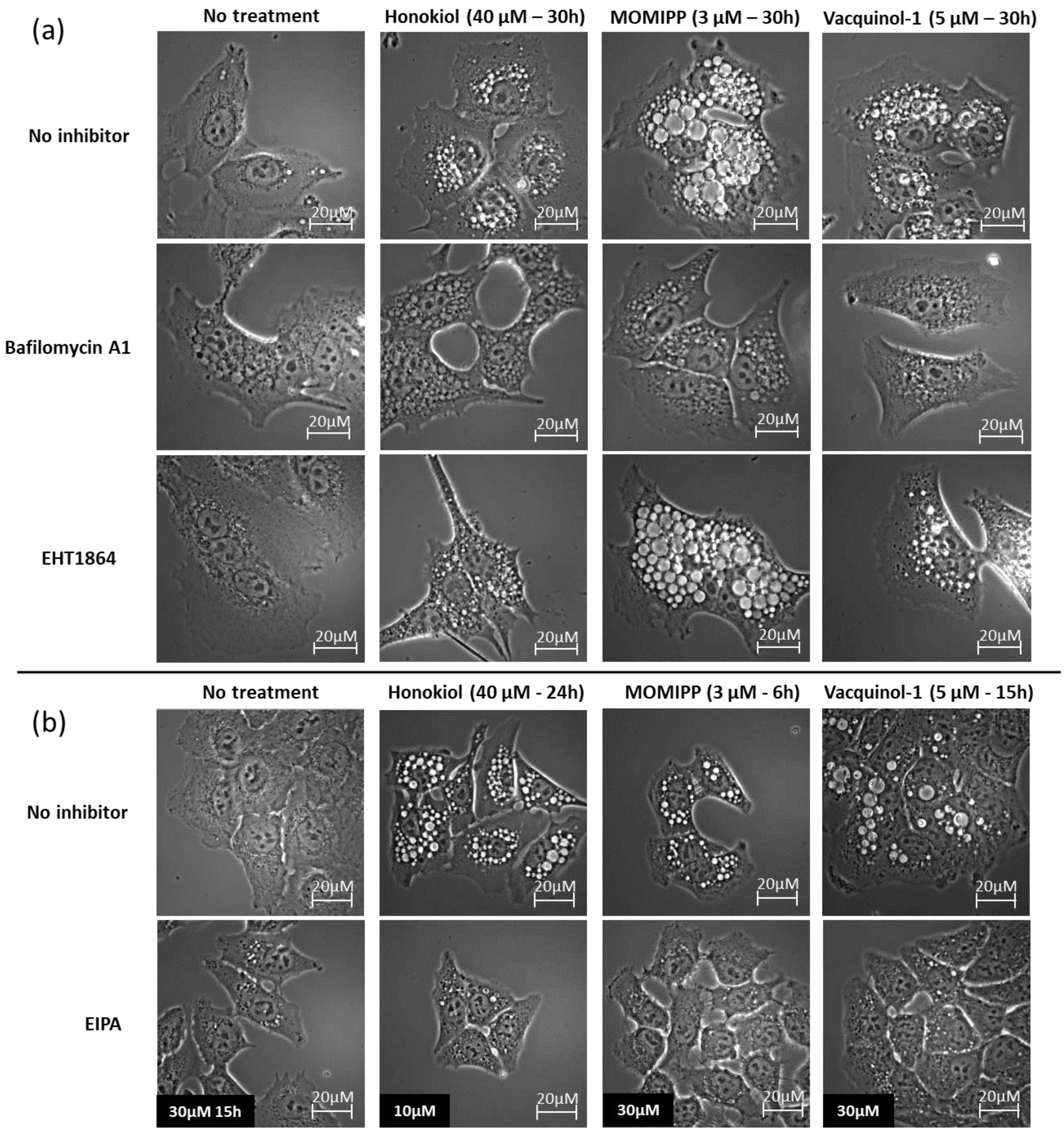

Figure S3. Effects of various inhibitors on vacuoles formation following honokiol, vacquinol-1 and MOMIPP treatment in Hs683 cells. Representative brightfield illustrations of Hs683 cells treated as follows: (**a**) pre-treated 1 h with bafilomycin A1 (100 nM) before honokiol (40 µM), MOMIPP (3 µM) or vacquinol-1 (5 µM) treatment, co-treatment with EHT1864 (25 µM) and the compound. Bafilomycin A1 inhibits the refringent vacuoles induced by each compound but EHT1864 has no effect on vacuoles. (**b**) Cells were co-treated with EIPA (10 and 30 µM) and each compound. EIPA inhibits vacuoles induced by honokiol, MOMIPP and vacquinol-1. The experiment has been conducted two times in triplicate.

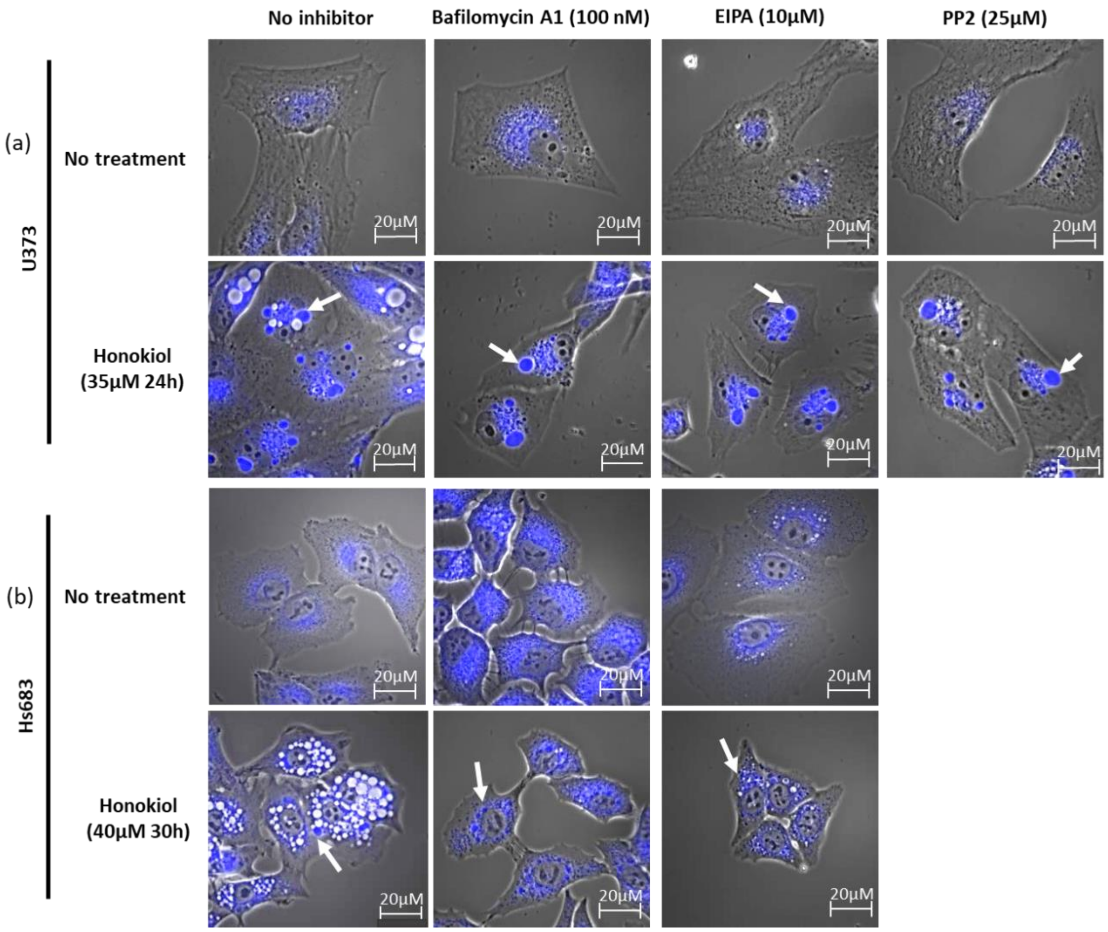

Figure S4. Illustration of the ER-Tracker staining of U373 (**a**) and Hs683 (**b**) in presence of honokiol with or without the various vacuolization inhibitors. ER-positive vacuoles (white arrows) induced by honokiol are not inhibited by the vacuolization inhibitors bafilomycin A1 (100 nM 1 h), EIPA (10 µM) or PP2 (25 µM). The experiment has been conducted two times in triplicate.

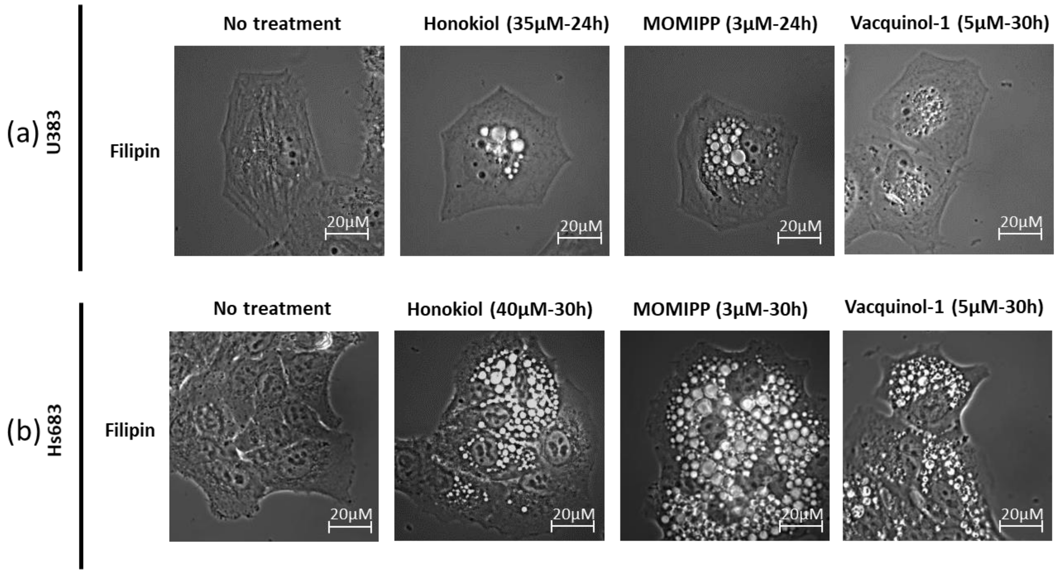

Figure S5. Effects of filipin on vacuoles formation following honokiol, vacquinol-1 and MOMIPP treatment in U373 (**a**) and Hs683 (**b**) cells. Representative brightfield illustrations of U373 (**a**) and Hs683 (**b**) cells treated as indicated in the presence of filipin at 1 µg/mL (pre-treatment of 30 min before addition of honokiol, vacquinol-1 or MOMIPP treatment). No inhibition of the vacuole

formation could be obtained at that filipin concentration. The experiment has been conducted two times in triplicate.

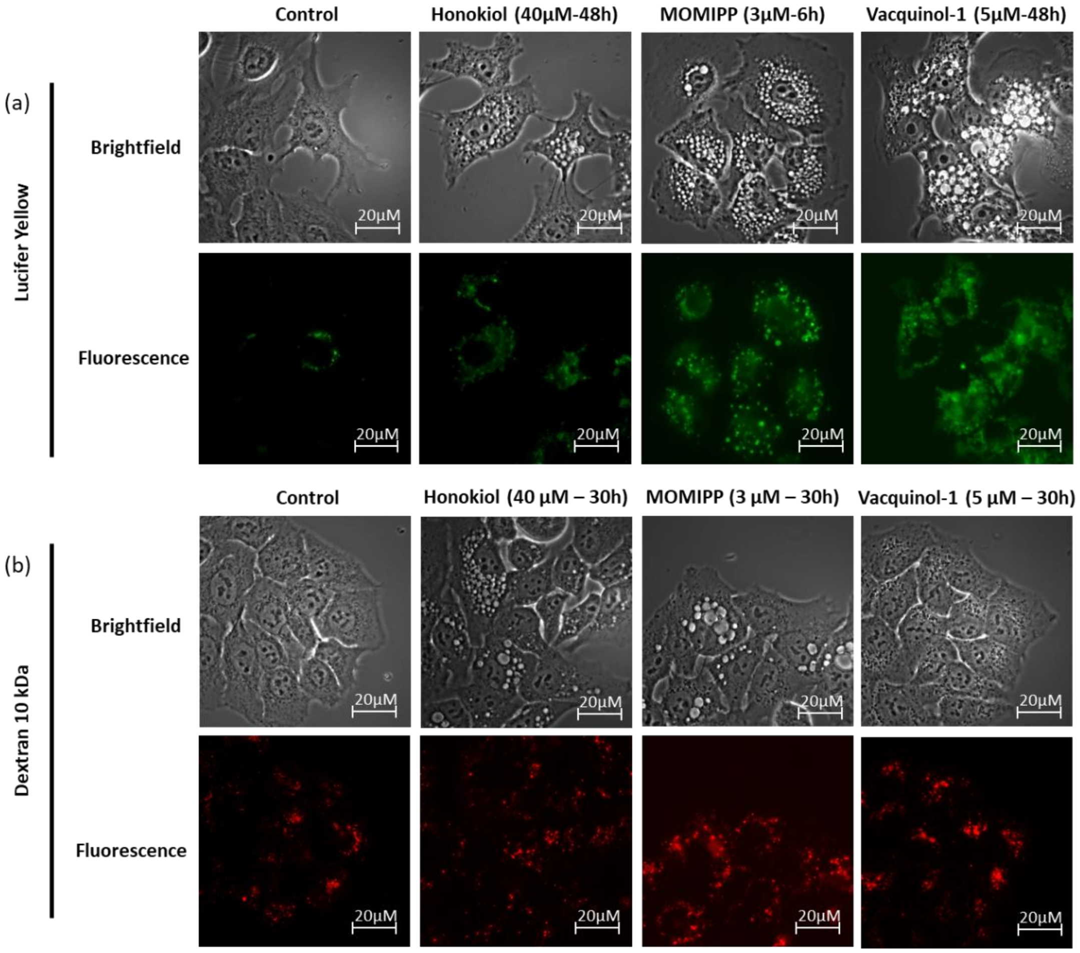

Figure S6. Illustration of lucifer yellow (**a**) and fluorescent 10 kDa dextran (**b**) uptake in Hs683 cells treated with honokiol, MOMIPP or vacquinol-1. Cells were treated with each compound in the presence of lucifer yellow (100 µg/mL) or 10 kDa Texas-Red labeled dextran (125 µg/mL). Exposure time is the same for all conditions with lucifer yellow, but it has been adjusted for each condition with 10 kDa dextran. The experiment has been conducted three times in duplicate for lucifer yellow and, twice in duplicate for 10 kDa dextran.

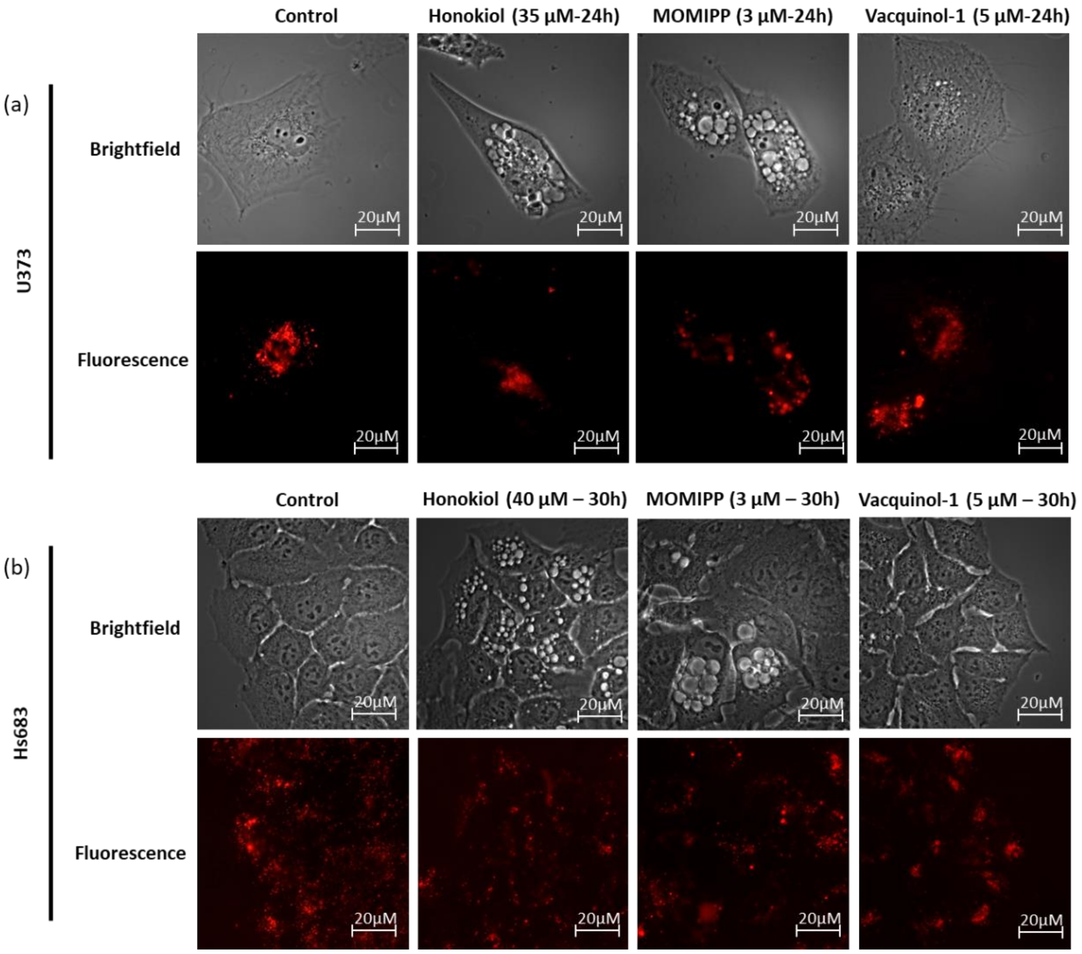

Figure S7. Illustration of fluorescent 70 kDa dextran internalization in U373 (**a**) and Hs683 (**b**) cells treated with honokiol, MOMIPP, vacquinol-1. Cells were treated with each compound in the presence of 70 kDa Texas-Red labeled dextran (125 µg/mL). Exposure time is the same for all conditions. The experiment has been conducted twice in duplicate.

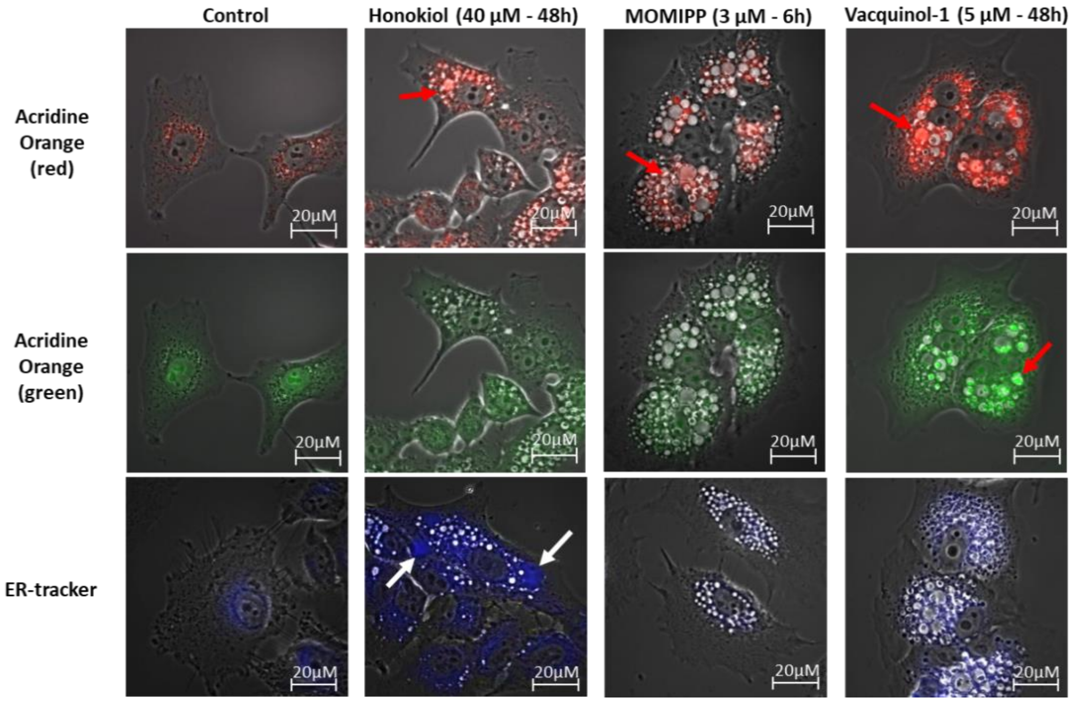

Figure S8. Illustrations of the fluorescent characterization of the vacuoles using different fluorescent cell compartment trackers in Hs683 cells. Two kinds of vacuoles are observed after treatment with honokiol: refringent vacuoles stained by acridine orange (red arrows) and dark dense vacuoles stained by the ER-tracker (white arrows). Some vacuoles induced by MOMIPP are red-positively with acridine orange (red arrow) but none to the ER-tracker. Vacquinol-1 induced vacuoles are red-positively (red arrow) but also green-positively stained by acridine orange (red arrow). This experiment has been conducted three times in duplicate.

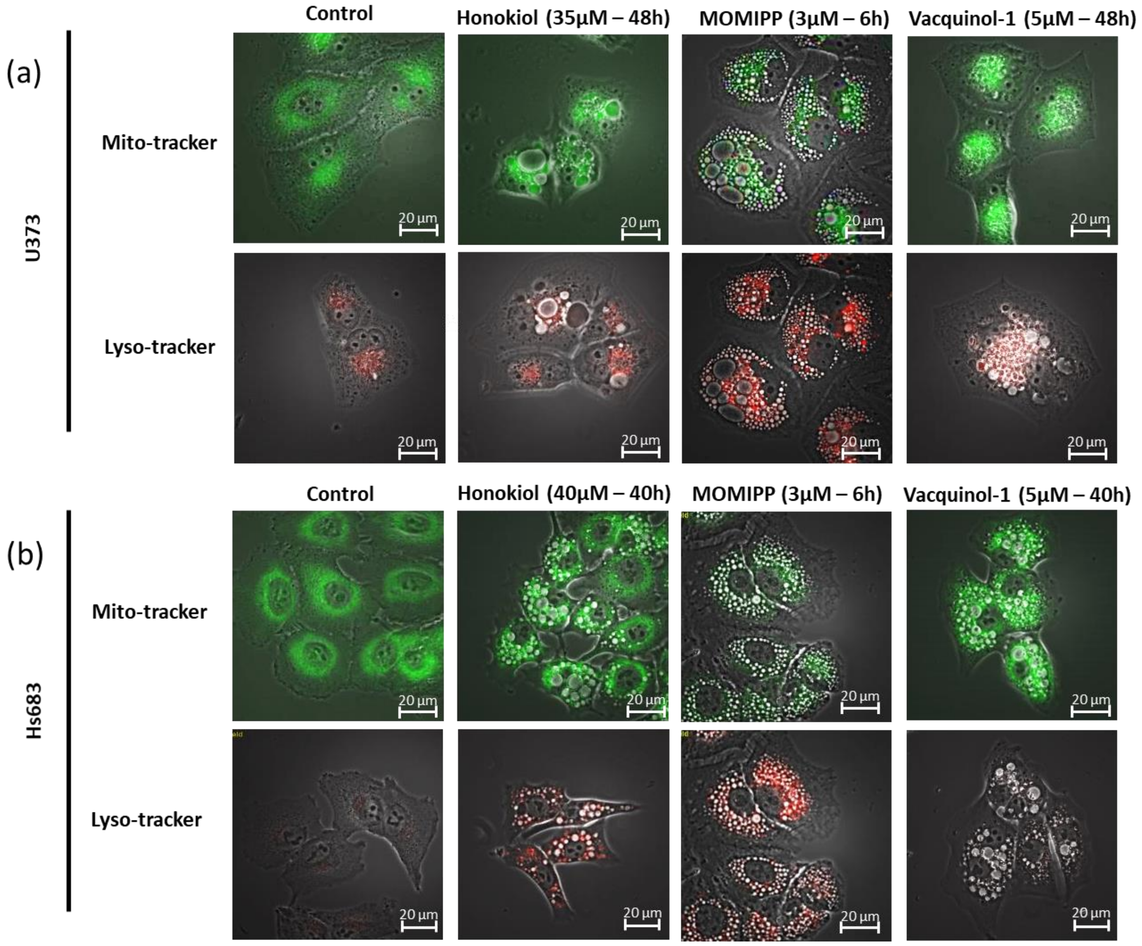

Figure S9. Illustration of the fluorescent marking of the vacuoles using mitochondrial and lysosomal cell compartment trackers in U373 (**a**) and Hs683 (**b**) cells. Vacuoles induced by the three compounds are negative to the mitochondrial marker. Some vacuoles induced by honokiol and MOMIPP are positive to the lysosomal tracker. The experiment has been conducted three times in duplicate.

© 2019 by the authors. Submitted for possible open access publication under the terms and conditions of the Creative Commons Attribution (CC BY) license (http://creativecommons.org/licenses/by/4.0/).
